# The Unmet Needs of Lysosomal Storage Disorders from Early Diagnosis to Caregiving Pathways: An Italian Perspective

**DOI:** 10.3390/jcm13226981

**Published:** 2024-11-20

**Authors:** Giancarlo Castaman, Silvia Linari, Antonio Barbato, Niko Costantino, Carlo Dionisi-Vici, Francesca Menni, Elena Procopio, Silvia Ramat, Fernanda Torquati, Elena Verrecchia, Maurizio Scarpa

**Affiliations:** 1 Center for Bleeding Disorders and Coagulation, Department of Oncology, Careggi University Hospital, 50134 Florence, Italy; linaris@aou-careggi.toscana.it; 2 Department of Clinical Medicine and Surgery, “Federico II” University Hospital, 80131 Naples, Italy; abarbato@unina.it; 3 Head of Public Affairs, Cometa ASMME (Association for the Study of Inherited Metabolic Disorders), 35020 Padua, Italy; n.costantino@cometaasmme.org; 4 Division of Metabolic Diseases and Hepatology, Ospedale Pediatrico Bambino Gesù IRCCS, 00165 Rome, Italy; carlo.dionisivici@opbg.net; 5 Fondazione IRCCS Ca’ Granda Ospedale Maggiore Policlinico, Regional Clinical Center for Expanded Newborn Screening, 20122 Milan, Italy; francesca.menni@policlinico.mi.it; 6 Meyer Children’s Hospital IRCCS, 50139 Firenze, Italy; elena.procopio@meyer.it; 7 Parkinson Unit, Neuromuscular-Skeletal and Sensory Organs Department, AOU Careggi, 50134 Firenze, Italy; silvia.ramat@unifi.it; 8 President of the Italian Gaucher Association and Member of the European Gaucher Alliance, 50066 Reggello, Italy; info@gaucheritalia.org; 9 Periodic Fever and Rare Diseases Research Centre, Catholic University of Sacred Heart, 00168 Rome, Italy; elena.verrecchia@policlinicogemelli.it; 10 Regional Coordinator Centre for Rare Diseases, University Hospital of Udine, 33100 Udine, Italy; maurizio.scarpa@asufc.sanita.fvg.it

**Keywords:** lysosomal storage disorder, diagnostic delay, newborn screening, transition, territorial management, multidisciplinarity

## Abstract

**Background/Objective:** Lysosomal storage diseases (LSDs) are a group of rare, inborn, metabolic errors characterized by deficiencies in normal lysosomal function and by the intralysosomal accumulation of undegraded substrates, resulting in the damage of multiple organ systems. The spectrum of clinical manifestations is extremely heterogeneous. LSD diagnosis and management still present many issues. **Methods:** A group of Italian experts and patients’ representatives met to discuss some critical aspects, and among the most impactful are early diagnosis, the transition of the patient from pediatric to adult age, territorial management, and the multidisciplinary approach. **Results:** Possible solutions to diagnostic delays may be a widespread newborn screening and screening programs on selected populations. The lack of a structured transition process could be helped by the drafting of shared diagnostic and therapeutic care pathways beyond the availability of databases accessible to the different levels that manage a patient. Territorial management could benefit from telemedicine, but a homogeneous diffusion of home therapy, not yet everywhere possible, is essential. A fundamental role is played by the patient associations, which should be increasingly involved in the political choices. It is also crucial to create structured multidisciplinary teams of experts for disease management and comorbidities. A transversal need appears to be greater training on LSDs. In Italy, the “Statement of Udine” was developed to guide further steps towards improvements in inherited metabolic medicine in adults, referencing the experience from the United Kingdom. **Conclusions:** Much can be done for the early diagnosis and management of LSDs with an effective treatment, but many aspects need improvement for the overall management of the patient. An investment in dedicated resources, formal recognition, and training is needed to address these unmet needs.

## 1. Introduction

Lysosomal storage disorders (LSDs) are a group of more than 60 genetically determined diseases caused by deficiencies in lysosomal enzymes, membrane transporters, or other proteins involved in lysosomal biology. Lysosomal dysfunction resulting from the deficiency of a single enzyme necessary for the metabolism of lipids, glycoproteins or mucopolysaccharides, or molecule transport enzymes located on the lysosomal membrane and at the cytoplasmic level leads to the accumulation of the unmetabolized substrate within the lysosomes themselves. This accumulation causes the loss of cellular functions and systemic alterations that can involve multiple organs and systems [1,2]. The resulting symptoms are very heterogeneous and vary depending on the pathology. LSDs are classified according to the type of material that accumulates (lipid storage disorders, mucopolysaccharides, glycoproteins) [3] (Table 1).

Hereditary inheritance is autosomal recessive, apart from Anderson–Fabry disease, Danon disease, and Mucopolysaccharidosis (MPS) type II (Hunter disease), which have X-linked inheritance. Individually, LSDs are rare [4,5] (Table 2), while globally, their incidence has been estimated at 1 in 7000 to 1 in 8000 live births. Most LSDs are characterized by a progressive course, the accumulation of the unmetabolized substance may begin during early embryonic development, and the clinical presentation may range from an early, severe phenotype to a slower-progression, late-onset disease. The spectrum of symptoms is very heterogeneous and can include severe intellectual disabilities, cardiac abnormalities, visceromegaly, bone deformities, muscle weakness, respiratory failure, visual defects, and skin changes.

The diagnosis of most LSDs is primarily based on the detection of the specific enzyme deficiency. In these cases, molecular genetic testing can confirm the enzyme diagnosis. Once the genotype of an individual LSD patient has been ascertained, genetic counseling should include predicting the possible phenotype and identifying carriers in the at-risk family. Unfortunately, the genotype/phenotype correlation is not always evident for most LSDs, and therefore, it is difficult to predict the evolution of the disease in the patient. As shared with other rare diseases, various problems are still present today that lead to a series of difficulties for the appropriate management of these patients. While in Italy, there is no specific document addressing these problems in LSD, a recent paper reviewed the challenges faced by patients with rare disease across the world [6]. In Europe, delayed diagnosis, disparities in available medical treatment across countries, access to treatment, and reimbursement issues were identified as crucial aspects among them [6].

With this as background, a group of Italian experts and patients’ representatives met to discuss some critical aspects in the diagnosis and management of LSDs. Among the most impactful are early diagnosis, the transition of the patient from pediatric to adult age, territorial management, and the multidisciplinary approach. All the participants agreed on the topics and the final content results of their analysis and discussion.

## 2. The Problem of Diagnosis

Diagnostic delay is a common problem for LSDs that occur in adulthood. In fact, pediatricians are more familiar with the severe “classic” variants that occur in childhood, while adult physicians often rarely consider late-onset forms in their differential diagnosis. It has been shown that 1 in 6 patients with Gaucher disease reported a diagnostic delay of 7 years or more from the first medical consultation [7], while for the diagnosis of Fabry disease, an average diagnostic delay of 13.7 years is reported in men and 16.3 years in women [8]. Diagnostic delays in LSD can result in irreversible damage. In Gaucher disease, for example, these typically occur at the level of bone and liver, growth retardation, and the execution of inappropriate diagnostic–therapeutic procedures (splenectomy, liver biopsy) may occur [9,10].

Early diagnosis is, therefore, essential, particularly for LSDs, for which there is an effective therapy and newborn screening can play a decisive role, representing an important secondary health prevention intervention. The goal of newborn screening programs is, in fact, to promptly diagnose congenital diseases for which specific therapeutic interventions are available that, if undertaken before the onset of symptoms, can significantly improve the prognosis and outcomes of the disease, as well as the quality of life of patients and caregivers. Italy is the European country with the most advanced newborn screening policy, as defined pursuant to Law 167/2016 and subsequent updates and implementing decrees, currently including over 40 hereditary metabolic diseases, including, in some regions (Friuli-Venezia Giulia, Veneto, Piedmont, Liguria, Tuscany, Lazio, Abruzzo, and Campania) as a pilot project, some LSDs, although the institutional process for the approval of the inclusion of Pompe disease, Fabry disease, Gaucher disease, and mucopolysaccharidosis type 1 is still ongoing. The inclusion of newborn screening for rare metabolic diseases in the new Essential Levels of Care (LEAs) makes it possible to guarantee screening to all newborns. However, there is no uniformity in the screening system across the country, and the LSDs investigated are still defined at the regional level and based on pilot projects. In Veneto and Friuli-Venezia Giulia, for example, newborn screening is carried out for Pompe disease, Fabry disease, Gaucher disease, mucopolysaccharidosis type 1, and Niemann–Pick A/B disease; in Campania, on the other hand, Pompe disease, Fabry disease, Gaucher disease, and mucopolysaccharidosis type 1 are investigated, while in Tuscany, only Pompe disease, Fabry disease, and mucopolysaccharidosis type 1 are investigated. There are several considerations related to newborn screening: first of all, the need for an operational protocol for the management of the screenings themselves, with a clear definition of the methods of management of the patient who has tested positive. In addition, it becomes essential to perform further confirmatory diagnostic tests and, above all, the subsequent management of the newborn by the clinical center of reference, in order to promptly start the specific treatment and follow the follow-up required for the disease. Newborn screening should, therefore, be understood not only as a laboratory diagnosis, but above all as a primary clinical management system of the newborn with LSD. There are also no guidelines for the medical management of individuals who are positive for newborn screening, but are asymptomatic; a shared directive of this aspect becomes necessary, also in relation to the sustainability of the National Health System (NHS).

Clearly, newborn screening also involves a non-negligible ethical–psychological burden, such as anxiety and uncertainty poured first on the parents, then on the patient due to the presence of a diagnosis of illness before the appearance of any clinical manifestation, which may never even occur.

National guidelines are necessary that will define, in a homogeneous way, the methods of performing newborn screening, which represents one of the most advanced tools for the prevention of congenital diseases, complex and multidisciplinary, and has important public health implications involving health professionals, people with rare diseases, families, and associations. A diagnostic possibility and a consequent uniform management guarantee the citizen a fair offer for access and treatment wherever he or she resides.

The situation is different for LSD screening in populations selected on the basis of the presence of initial signs and/or symptoms characteristic of the type of disease; in at-risk populations, erroneous and inappropriate diagnoses and therapies are avoided, avoiding unnecessary and/or invasive procedures and tests. Several screenings on selected populations are currently underway as multicenter studies, but globally shared criteria should be defined to enhance this modality of early diagnosis [11,12,13,14,15].

An important aspect to effectively support the early diagnosis of LSD and its management is represented by training and informing specialists who may come into contact with potential patients, with the aim of inducing them to differentiate the type of lysosomal disease. It could be useful to introduce the topic of rare diseases into university education, not only for clinical training, but also for relational training. The mode of communication in chronic genetic diseases is a non-negligible aspect; knowing how to communicate with a family or an adult patient during the different stages of the disease, from diagnosis to management or the appearance of a complication, should be a must-have. 

## 3. The Transition from Pediatric to Adult Age

The need to train health care professionals on rare diseases is also closely linked to the issue of taking charge of patients in the transition [16,17]. The transition represents the process of maturation of the patient from pediatric to adult age, with a consequent transfer of management from the hospital pediatrician to the internist. It is essential that the internist has not only specialized skills on LSD and its evolution, but also relational listening and reception, to manage the transition from communication to parents to direct communication with the young patient. In the transition, there are different moments that the doctor must be able to recognize and accompany, facilitating problematic transitions not only from childhood to adulthood, but also to old age and frailty. 

The transition is also an important moment of communication between professionals, as a precise flow of information must take place. This can be a delicate, complex, and often not precisely structured or institutionalized aspect. It would be desirable to create complete databases accessible to the different levels that manage the patient (different hospitals, hospitals–territories, different regions). In settings where pediatrics and internal medicine are in the same hospital setting, managing the transition is easier, both for specialists and patients. The drafting of diagnostic and therapeutic care pathways (PDTAs) can also be useful in managing the transition. 

In the field of rare diseases and LSDs, a geographical transition is also frequent, which occurs when the patient is forced to move from the region to another for management and treatment. Sometimes the patient may not have received clear information about the possibility of management near their residence. Therefore, clarity of information and ease of retrieval are desirable.

Also, on the issue of transition, it is clear that health economics policies are needed that invest in the training of young doctors and in the continuity of care of patients.

Regarding the Italian Situation, in 2022, a collaboration between the adult metabolic working group of the Italian Society for the Study of Inherited Metabolic Disorders and Neonatal Screening (SIMMESN) and the European Reference Network for Hereditary Metabolic Disorders (MetabERN) was established to face problems linked to inherited metabolic disorders (IMDs) in adulthood. “The Statement of Udine” was developed to guide further steps towards improvements in inherited metabolic medicine in adults, referencing the experience from the UK [18]. The aim of this paper is to present “The Statement of Udine,” explaining its background and its possible applications. The paper did conclude that the knowledge of the management of these patients should quickly increase and improve in the next few years, and the creation of a new branch of adult medicine dedicated to IMDs is advocated. Since the number of adults diagnosed with IMDs is increasing, several issues should be addressed in the near future, including the lack of adult specialists confident in the management of these diseases, the lack of standardized and efficient transition programs, and the need to create adult services working in multidisciplinary teams to manage multisystemic disorders [18]. This document should guide the next steps for the development of the management of adults with IMDs in Italy, and it may be useful for other countries that are facing analogous problems.

## 4. Territorial Management

The territorial management of LSD patients presents several critical issues: first of all, the need to improve the relationship with pediatricians of free choice and general practitioners. They rarely take care of patients and tend to refer LSD patients to the specialists of the Reference Center even for reasons not related to the pathology or in any case of little importance. Here, too, a lack of specific knowledge can be the cause of the problem. As a solution to this, the positive experience of the Milan Center is reported, which, using telemedicine, has promoted meetings between specialists, local doctors, and families of affected people with the aim of managing children in their environments.

Assistance on the ground is lacking in several respects, from the provision of devices to the offer of services such as the rapid activation of home care or physiotherapy, which is essential for several LSDs. These deficiencies in the system are too often transformed into expenses borne by the family, with the creation of an inconsistent gap between the provision of very expensive therapies by the NHS and the lack of an adequate care response in the area. 

In Italy, the average annual cost of expenditure for a patient with Fabry disease, for example, is EUR 142,387, of which EUR 130,445 is attributable to the cost of enzyme replacement therapy and its administration. Furthermore, an evaluation of patients with type 2 MPS shows that the administration of replacement therapy in a hospital environment is more expensive by about EUR 7000/patient/year, not only because of the necessary travel and the loss of productivity of the patient or caregiver, but also because of the way the therapy is administered. The implementation and homogenization of home therapy, therefore, has several advantages, not only economic, but above all in terms of improving the quality of life [19,20]. 

The problem of territorial management is not only related to clinical needs, but also to social ones, as there is often a lack of effective social, occupational, and scholastic inclusion of patients with physical and/or intellectual disabilities. These, therefore, often remain the responsibility of the families, and their only current possibility of different placement is institutionalization. The burden of care for these patients is the prerogative of families, who, in addition to being burdened by the care and management aspects, also must face a significant economic and emotional commitment. All this inevitably creates a difference in treatment, linked to the economic and cultural situation of the family to which it belongs. Social policies, in addition to the enunciation of law, should be concerned in practice with the management of these unmet needs. For the purpose of social inclusion, LSD training should also be addressed to educational institutions to promote the correct integration between social/health/inclusion first in school, then in the world of work. Even in the latter area, there are significant difficulties, as there is a lack of specific safeguards to manage absences from work due to medical examinations and therapies.

Associations are certainly the main resource for families and patients; the sharing of needs and problems is often the system that allows us to intercept requests for help. In addition, associations are the main resource for the circulation of information. Although there are support and service networks in the area, information and access methods are often not easily available, and even health personnel are sometimes unaware of them. Also, the training of health personnel on the existence of territorial resources and dissemination of the related options is necessary. 

## 5. Multidisciplinarity

Chronic diseases, and rare ones in particular, require a progressive modification of management by the doctor. If in the first phase, the approach is mainly aimed at diagnosis and the search for an effective pharmacological therapy, in the second phase, solutions are sought to improve the patient’s functionality and allow him or her a fair social participation and a good quality of life. In both phases, but especially in the second, the best approach is the multidisciplinary one. This approach places the patient at the center of the care process and is part of what is called the “bio-psycho-social model.” In this model, the person is no longer seen as a “patient,” but as an individual who expresses his or her being in ways that are dependent not only on biological factors (health or disease), but also on interaction with social elements (e.g., family and friends) and personal psychological factors.

**Figure 1:**
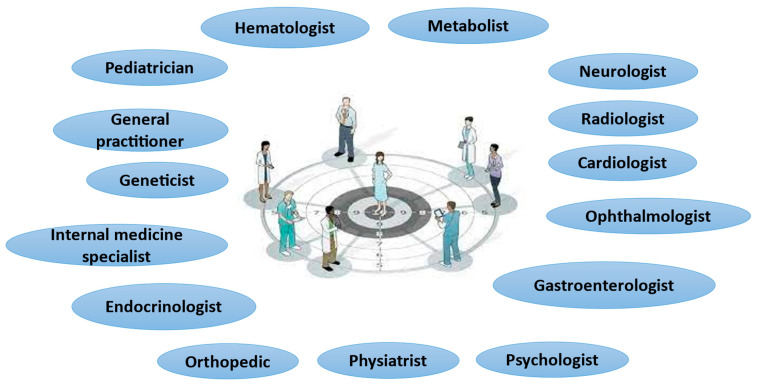
Multidisciplinary management of lysosomal accumulation diseases.

The management of the LSD patient, therefore, involves collaboration between different professionals both horizontally (in the same phase of life) and vertically (in the transitions between the different phases). Over time, hospital pediatricians, internists, pediatricians, general practitioners, psychologists, nurses, physiotherapists, and different specialists based on single LSD have to interface, engage in a dialogue, and modulate themselves (Figure 1). Multidisciplinarity is also essential for the diagnosis and management of comorbidities that may arise in patients with LSD and that are not necessarily related to the underlying disease. For optimal multidisciplinary management, a “case manager,” preferably covered by a doctor, would be very useful to coordinate the different professional figures. The creation of multidisciplinary teams should be a shared and possibly formalized path and not based on simple occasional relationships between specialists depending on the problem to be managed. It goes without saying that the rarity of these disorders, the associated costs, and the high degree of skills required determine the need to identify multidisciplinary centers and teams at the level of individual regions, where possible. Following the example of the Multidisciplinary Oncology Groups, i.e., working groups that meet at regular intervals made up of specialists from various areas, it would also be desirable to create a multidisciplinary team (at various levels) for LSDs that takes charge of the different aspects of specific diseases, thus contributing to the definition and implementation of diagnostic pathways and the therapeutic and rehabilitative treatment of patients, in compliance with evidence-based medicine.

Increasingly, the care and treatment of patients are oriented by the assessment of their quality of life; therefore, the implementation of patient-reported outcome measures (PROMs) is also an important goal in LSDs [21,22,23,24].

## 6. Conclusions

LSDs represent a paradigm of rare diseases. Rare diseases, including those of genetic origin, are life-threatening, chronically debilitating, and of low prevalence.

Low prevalence means rare diseases affect a small portion of the population: less than 5 per 10000 people in the community (Decision No. 1295/1999/EC of the European Parliament and of the Council of 29 April 1999). The EU’s strategic objective for rare diseases is to improve patient access to diagnosis, information, and care. It assists in pooling scarce resources spread across the EU, enabling patients and professionals to share expertise and information. The European response aims to set up and support European Reference Networks (ERNs), develop and manage the European Platform on Rare Disease Registration (EU RD Platform), support the definition, codification, and inventory of rare diseases, support the designation and authorization of orphan medicinal products, expand the knowledge base through research and innovation, develop new therapies and diagnostic tools for rare diseases, enhance the FAIRification of rare diseases registries and data, improve the recognition and visibility of rare diseases, also at global level via the International Rare Diseases Research Consortium (IRDiRC), empower patient organizations, and promote the development of national rare diseases plans, strategies, and research. A major effort has been devoted to launch national plans for rare diseases. The Council Recommendation of 2009 on an action in the field of rare diseases, amongst other things, recommended that Member States establish and implement plans or strategies for rare diseases to aim to ensure that patients with rare diseases have access to high-quality care. These national plans and strategies can also help promote ERNs at national levels and facilitate the exchange of best practices among EU countries. The Joint Action JARDIN on the integration of the ERNs into national health care systems will also seek to achieve the sustainability of its proposed actions and implementations by their integration into updated national plans and strategies for rare diseases.

In Italy, Law 279/2001 (updated 175/2021) on Rare Diseases, together with the network of Regional Reference Centers on Rare Diseases, provides the tools to deal with the above-mentioned unmet needs. Furthermore, to help in pursuing this goal, thanks to the EU cross-borders law of 2011, Italy is participating with more than 300 centers (including 18 dedicated to metabolic disorders) into the ERNs, which are now being jointly incorporated in the National Health System (JARDIN). The National Plan on Rare Diseases released in 2023 identifies priorities and main actions for an optimal management of rare diseases. Thus, LSDs may represent an important setting to assess the implementation of these guidelines and recommendations. In conclusion, much can be done today for the early diagnosis and management of LSDs that have an effective treatment, but there are just as many aspects that need improvement for the overall management of the patient (Table 3). An investment in dedicated resources, formal recognition, and training is needed to address the unmet needs of LSD patients and their families.

## Figures and Tables

**Table 1:** Classification of lysosomal storage disorders.

Disease	Affected Pathway	Clinical Example
Sphingolipidoses	Degradation of ceramide-containing membrane lipids	Gaucher disease, Fabry disease, Acid sphingomyelinase deficiency
Mucopolysaccharidoses	Glycosaminoglycane degradation	MPS type I (Hurler disease)
Oligosaccharidoses	Degradation of complex carbohydrate side-chains of glycoproteins	a-Mannosidasis
Mucolipidoses	Deficiency of several lysosomal enzymes, many pathways affected	Mucolipidosis type II
Lipid storage disease	Degradation of lipid compounds other than sphingolipids	Cholesterol ester storage disease
Glycogen storage disease type ii	Intralysosomal glycogen breakdown due to deficiency of lysosomal acid maltase	Pompe disease
Lysosomal transport defects	Failure to transport certain compounds across the lysosomal membrane	Sialic acid transport defect

**Table 2:** Global epidemiological distribution of some lysosomal storage disorders.

Disease	Incidence	Prevalence
Cystinosis	1/281,000	1/192,000
Anderson–Fabry disease	1/117,000	1/117,000
Gaucher disease	1/59,000	1/57,000
GM_1_ gangliosidosis	1/422,000	1/384,000
Krabbe disease	1/201,000	1/141,000
α-mannosidosis	1/1,056,000	1/1,056,000
MPS I	1/111,000	1/88,000
MPS II	1/162,000	1/136,000
Acid sphingomyelinase deficiency	1/264,000	1/248,000
Niemann–Pick C	1/211,000	1/211,000
Pompe disease	1/201,000	1/146,000
Sandhoff disease	1/422,000	1/384,000
Tay–Sachs disease	1/222,000	1/201,000
Wolman disease	1/704,000	1/528,000
Metachromatic leukodystrophy	1/100,000	1/100,000
All LSDs	1/9000	1/7700

**Table 3:** Unmet needs in lysosomal storage diseases and their possible solutions.

Unmet Need	Main Problems	Possible Solution
Diagnostic delay	Worsening and progression of the disease	Newborn screening
	Long patient journey	LSD screening in populations selected
	Lack of genetic counselling	Increase awareness among health care professionals
Transition from pediatric to adult age	Geographical transition	Drafting of diagnostic and therapeutic care pathways (PDTAs)
	Loss of clinical information in the transition	Invest in the training of young doctors and in the continuity of care of patients
	Loss of therapeutic/care continuity	Complete databases accessible to the different levels that manage the patient
		Formation of structures where pediatric and adult doctors coexist
Territorial management	Relationship between Reference Center and primary care doctors	Improve the relationship with pediatricians of free choice and general practitioners
	Home care difficulty	National health system or company-supported home therapy
	Lack of knowledge in LSD	Telemedicine
	Patients referred to center for all medical problems	Telemedicine
	Lack of specific medical devices or physiotherapy	Patient associations included in political choice
	Social inclusion	Inclusive social policies
Multidisciplinarity	Management of the disease	Case manager–disease manager
	Management of comorbidities	Expert multidisciplinary team at regional level
	Quality of life evaluation	Patient-reported outcome measures

## Data Availability

No new data were created or analyzed in this study. Data sharing is not applicable to this article.
